# Mesenchymal Stem Cell–Conditioned Media Modulate HUVEC Response to H_2_O_2_: Impact on Gene Expression and Potential for Atherosclerosis Intervention

**DOI:** 10.1155/2024/7726493

**Published:** 2024-07-17

**Authors:** Abdulmajeed Alqasoumi, Mansour Alsharidah, Amer Mahmood, Mona Elsafadi, Osamah Al Rugaie, Khalid M. Mohany, Khalid A. Al-Regaiey, Khaleel I. Alyahya, Alaa A. Alanteet, Norah K. Algarzae, Hanan K. AlGhibiwi, Adel AlHomaidi, Mohammad Abumaree

**Affiliations:** ^1^ Department of Pharmacy Practice College of Pharmacy Qassim University, Qassim, Saudi Arabia; ^2^ Department of Physiology College of Medicine Qassim University, Qassim, Saudi Arabia; ^3^ Stem Cell Unit Department of Anatomy College of Medicine King Saud University, Riyadh, Saudi Arabia; ^4^ Department of Biology and Immunology College of Medicine Qassim University, Qassim, Saudi Arabia; ^5^ Department of Medical Biochemistry Faculty of Medicine Assiut University, El Gamma Street, Assiut City 71515, Egypt; ^6^ Department of Physiology College of Medicine King Saud University, Riyadh, Saudi Arabia; ^7^ Department of Anatomy College of Medicine King Saud University, Riyadh, Saudi Arabia; ^8^ Department of Pharmacology and Toxicology College of Pharmacy King Saud University, Riyadh, Saudi Arabia; ^9^ Department of Pathology College of Medicine Qassim University, Qassim, Saudi Arabia; ^10^ Stem Cells and Regenerative Medicine Cell Therapy and Cancer Research (CTCR) King Abdullah International Medical Research Center (KAIMRC) King Saud Bin Abdulaziz University for Health Sciences (KSAU) King Abdulaziz Medical City Ministry of National Guard Health Affairs (MNGHA), Riyadh, Saudi Arabia

**Keywords:** cell therapy, endothelial cell functional properties, MSCs, oxidative stress

## Abstract

**Background:** We studied the potential of human bone marrow–derived mesenchymal stem cell conditioned media (hBMSC CM) in protecting endothelial cell properties (viability, proliferation, and migrations) from the deleterious effects produced by the inflammatory environment of H_2_O_2_. Additionally, we investigated their impact on the endothelial cells' gene expression of some inflammatory-related genes, namely, TGF-*β*1, FOS, ATF3, RAF-1, and SMAD3.

**Methods:** Human umbilical vein endothelial cells (HUVECs) were cultured individually under three conditions: alone, with varying concentrations of H_2_O_2_, or with varying concentrations of H_2_O_2_ and hBMSC CM. HUVEC adhesion, proliferation, and migration were evaluated using the xCELLigence system. The HUVECs' gene expressions were evaluated by real-time polymerase chain reaction (RT–PCR).

**Results:** Generally, we observed enhanced HUVEC viability, proliferation, and migration when cultured in media supplemented with H_2_O_2_ and hBMSC CM. Furthermore, the CM modulated the expressions of the studied inflammatory-related genes in HUVECs, promoting a more robust cellular response.

**Conclusion:** This study has illuminated the protective role of hBMSC CM in mitigating the damaging effects of H_2_O_2_ on endothelial cell function. Our data demonstrate that hBMSC CM enhances the viability, proliferation, and migration of HUVECs even under oxidative stress conditions. Additionally, the conditioned medium was found to modulate the gene expression of pivotal markers related to inflammation, suggesting a favorable influence on cellular response mechanisms.

## 1. Background

Endothelium, the innermost layer of blood vessels, plays a pivotal role in vascular homeostasis. The proper function of endothelial cells is crucial for regulating blood flow, maintaining vessel integrity, and mediating inflammatory responses [1]. Endothelial dysfunction is a hallmark of atherosclerosis, characterized by an impaired endothelial barrier, heightened proinflammatory state, and abnormal vasoreactivity [2, 3].

Oxidative stress is widely recognized as a key factor contributing to endothelial dysfunction and the pathogenesis of atherosclerosis. Reactive oxygen species (ROS), such as H_2_O_2_, are normal byproducts of cellular metabolism. However, excessive ROS production leads to oxidative stress, potentiating inflammatory processes, and vascular damage. H_2_O_2_ disrupts endothelial function by altering protein activity and generating cytotoxic radicals (e.g., peroxynitrite and hydroxyl radicals) which, in turn, can damage cellular DNA, proteins, and lipids [4, 5].

The genesis of atherosclerosis involves the interplay between oxidative stress and inflammation. Oxidative stress triggers the inflammatory response by activating nuclear factor kappa B (NF-*κ*B), a transcription factor that regulates the expression of various inflammatory genes. This leads to the recruitment of immune cells to the endothelium, further releasing cytokines and chemokines, thereby exacerbating vascular inflammation, and promoting the development of atherosclerotic plaques [2, 3].

The therapeutic potential of mesenchymal stem cells (MSCs) in modulating inflammation and oxidative stress has been highlighted in cardiovascular disease models [6, 7]. Human bone marrow–derived MSCs (hBMSCs) have shown promise due to their ability to differentiate into multiple cell lineages and their capacity for robust proliferation and migration in response to stimuli. More importantly, hBMSCs secrete a range of bioactive molecules with immunomodulatory and proangiogenic effects [8, 9].

The current study focuses on the need to understand the protective effects of hBMSC conditioned media (CM) against H_2_O_2_-induced oxidative stress in endothelial cells. The experiments are designed to show how hBMSC CM influences the viability, proliferation, migration, and gene expression of HUVECs under oxidative stress. This study is aimed at evaluating the potential of hBMSC CM as a therapeutic modality for improving endothelial dysfunction in atherosclerosis by mitigating the effects of oxidative stress and consequently the inflammatory response. Through the analysis of the gene expression of critical inflammation-related markers including transforming growth factor-beta 1 (*TGF-β1*), activating transcription factor 3, proto-oncogene (*ATF3*), accelerated fibrosarcoma-1 (*RAF-1*), and suppressor of mothers against decapentaplegic 3 (*SMAD3*) to understand the molecular mechanism underpinning the protective role of hBMSC CM and its potential impact on the pathophysiology of atherosclerosis.

## 2. Material and Methods

### 2.1. Cultivation of hBMSCs

For culturing of hBMSCs, immortalized MSC cells were employed as a substitute for primary hBMSCs. These cells flourished in a Dulbecco Modified Eagle Medium (DMEM) supplemented with 0.25 mol/L D-glucose, 10% fetal bovine serum, 1× penicillin–streptomycin, and essential amino acids. Cell growth was closely monitored until they reached a density of 50,000 cells/mL and a confluence of 70%–80% [10, 11]. Cell culture medium was changed every 3 days.

### 2.2. Cultivation of the Human Umbilical Vein Endothelial Cells (HUVECs)

The HUVECs were isolated from umbilical cord. Umbilical veins were meticulously rinsed with phosphate-buffered solution (PBS) and subsequently treated with a PBS containing 6 mg/mL of collagenase type II for digestion (catalog number 17101-015, Life Technologies). Following a 25-min incubation at 37°C, the HUVECs were extracted and resuspended in complete endothelial cell growth medium (catalog number PCS-100-041™, ATCC, United States); the cell culture medium was changed every 3 days. These cells were then further cultured at 37°C. HUVECs with a purity surpassing 95%, obtained from Passages 3–5, were utilized for this research [12]. The current experiment included seven groups (Table 1).

In this study, HUVECs were treated with CM derived from hBMSCs to assess the CM's protective effects against oxidative stress. The CM was used at a concentration of 30%, based on prior research indicating effective dosages for paracrine-mediated protection. The treatment was conducted over a period of 72 h, aligning with established protocols for observing CM-induced cellular responses. This timeframe was chosen to capture the dynamics of cell adhesion, proliferation, and migration under stress conditions induced by H_2_O_2_. The methodology was designed to reveal how MSC-derived factors within the CM contribute to the resilience and repair mechanisms of endothelial cells, a critical aspect of vascular health. It is to be mentioned that before exposing HUVECs to CM, the latter was centrifuged to get rid of debris.

### 2.3. Assessment of HUVEC Adhesion, Proliferation, and Migrations Using the xCELLigence System

For each experiment approximately 5 × 10^3^ HUVECs were seeded using a complete HUVEC growth medium. The cells were then cultured in 16-well culture plates (E-Plate 16, Roche Diagnostics, catalog number 05456048701) in a medium containing H_2_O_2_ concentrations of 100, 250, and 500 ng/mL. The culture was placed in the xCELLigence system (Roche Diagnostics, Mannheim, Germany) and incubated at 37°C. The HUVEC index (number of cells) was continuously monitored throughout the experiment [12].

The xCELLigence system enabled real-time assessment of HUVEC adhesion and proliferation. By modulating H_2_O_2_ concentrations, the xCELLigence system continuously monitored cell behavior through electrical impedance measurements. Briefly, 100 *μ*L of complete endothelial cell growth medium was added to wells in 16-well plates (catalog number 05469813001, E-Plate 16, from Roche Diagnostics). The baseline impedance was determined.

Subsequently, 5 × 10^3^ HUVECs were seeded in triplicate, either precultured with hBMSCs and H_2_O_2_ or cultured alone, and were seeded into the wells with 100 *μ*L of the HUVEC growth medium. After incubating the plates at room temperature for 30 min, equilibrium was achieved. For data collection, the culture plates were placed in the xCELLigence system and maintained at 37°C within a cell culture chamber. HUVEC index was automatically monitored over a 72-h period. The accompanying xCELLigence software (version 1.2.1) facilitated data interpretation. Cell adhesion data was collected 2-h postseeding, and the resulting cell index is presented as the mean ± standard error [10–12].

For cell proliferation assessment, the data is presented as the mean ± standard error of the cell index, adjusted based on the 2-h (postseeding) cell index. Proliferation rates were determined by examining the adjusted cell index at intervals of 24, 48, and 72 h. This experimental protocol was consistently applied for each trial. This approach enabled a comprehensive investigation of hBMSCs ± H_2_O_2_'s effects on HUVECs over a defined period [10–12].

Also, the system continuously monitored HUVECs' migration, while HUVECs alone served as controls in the upper chamber of the other groups. At the 24-h mark, the migration data was represented as cell index values. Migration was observed in 30% of the FBS positive control and 10% of the FBS negative control.

### 2.4. Analyzing HUVEC Gene Expression by Real-Time Polymerase Chain Reaction (RT–PCR)

Following a 24-h treatment of HUVECs with hBMSCs ± varying concentrations of H_2_O_2_, the expression profiles of TGF-*β*1, FOS, ATF3, RAF-1, and SMAD3 were evaluated. RNA was extracted from the HUVECs and subsequently employed for cDNA synthesis. The RT–PCR was performed three times using the Bio-Rad detection system (California, United States). The acquired data was interpreted and to represent the findings as a fold change using 2^−*ΔΔ*ct^ values. GAPDH was used as a housekeeping gene. The primers used during the experiment are shown in Table 2.

### 2.5. Statistics

All experiments were performed in triplicate; the error bars could not be added to the diagrams because the instruments did all the calculations and generated the graphs. The gathered data were systematically organized, categorized, and analyzed using the Kruskal–Wallis nonparametric test in SPSS software, version 22 (SPSS Inc., United States). Statistical significance was set at a *p* value less than 0.05.

## 3. Results

### 3.1. Real-Time Viability and Proliferation of HUVECs in Response to H_2_O_2_ Treatment ± hBMSC CM

**Figure 1:**
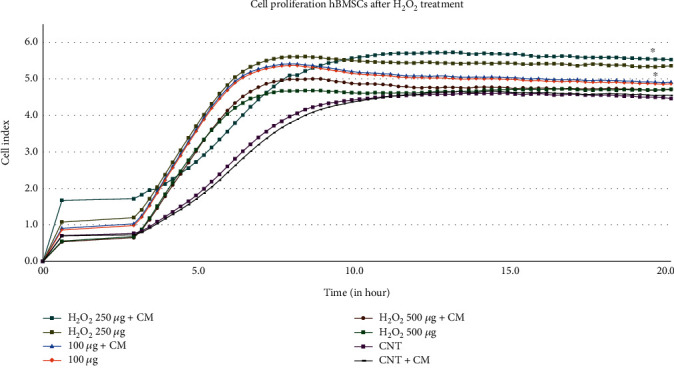
Real-time viability and proliferation of HUVECs in response to H_2_O_2_ treatment ± hBMSC CM. HUVECs were exposed to varying concentrations of H_2_O_2_ (100, 250, or 500 ng/mL) alone or together with pretreated hBMSC CM. The CNTs and the CNT + hBMSC CM are to be considered a baseline for comparison without the presence of H_2_O_2_. The bar graphs are presented as the mean ± SD of three independent experiments. ^∗^*p* < 0.05, ^∗∗^*p* < 0.01 vs. control + CM.

The presence of H_2_O_2_ exerted a significant impact on the proliferation of HUVECs. Interestingly, the application of 250 ng of H_2_O_2_ appeared to stimulate cell proliferation. Remarkably, this stimulatory effect was further enhanced when combined with hBMSC CM. In contrast, higher concentrations, such as 500 ng of H_2_O_2_, failed to induce a comparable level of increased proliferation, even in the presence of hBMSC CM. The control groups (CNTs) and CNT + hBMSC CM served as benchmarks for comparison, demonstrating the natural proliferation rate in the absence of H_2_O_2_ treatment (Figure 1).

### 3.2. HUVEC Proliferation Over 3 Days Under Various H_2_O_2_ Concentration Treatments

**Figure 2:**
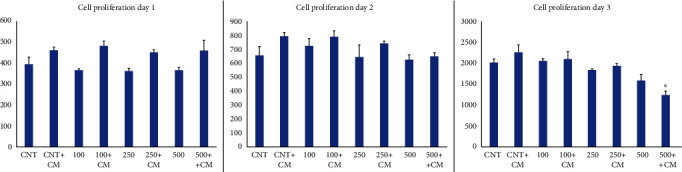
Comparative analysis of HUVEC proliferation over 3 days under various concentration treatments. HUVECs were treated for 3 days with different concentrations of H_2_O_2_ and monitored with or without hBMSC CM (all concentrations are in nanograms per milliliter). The bar graphs are presented as the mean ± SD of three independent experiments. ^∗^*p* < 0.05, ^∗∗^*p* < 0.01 vs. control + CM.

Figure 2 depicts the dynamic response of cells to various treatments over a 3-day period. The beneficial effect of hBMSC CM in enhancing cell proliferation in the presence of H_2_O_2_ is apparent, particularly in the 250 ng + hBMSC CM and 500 ng + hBMSC CM conditions. These findings provide valuable insights into the time-dependent effects of different H_2_O_2_ concentrations on cell proliferation, particularly in the presence of hBMSC CM.

Cell proliferation on Day 1 exhibited minimal differences across the various treatments. The baseline (CNT) group displayed a modest level of proliferation, with only marginal increases observed in the presence of CM (CNT + hBMSC CM) or varying concentrations of H_2_O_2_. Notably, the 250 ng + hBMSC CM treatment demonstrated the highest proliferation on Day 1.

The second day reveals a more pronounced difference in cell proliferation between the treatments. Samples treated with H_2_O_2_ 100 ng + hBMSC CM and 250 ng + hBMSC CM exhibit significantly higher cell counts. Notably, cell proliferation in samples treated with 500 ng without hBMSC CM is slightly lower than those treated with 250 ng of H_2_O_2_.

On the third day, an overall increase in cell proliferation across all conditions is evident. The 250 ng + hBMSC CM and 500 ng + hBMSC CM treatments demonstrate the highest cell proliferation. Notably, there is a significant jump in cell count in the 500 ng + hBMSC CM sample compared to its count on Day 2, suggesting a delayed but effective proliferative response to this treatment.

### 3.3. Differential Migration Responses of HUVECs to Various Concentrations of H_2_O_2_ in Real-Time Analysis

**Figure 3:**
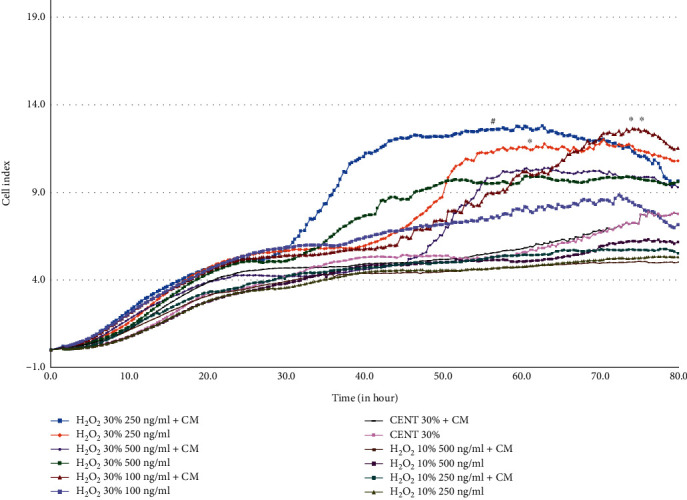
Differential migration responses of HUVECs to varying concentrations of H_2_O_2_ in real-time analysis. A real-time quantitative analysis investigated the migration of HUVECs upon exposure to different concentrations of H_2_O_2_, demonstrating the effects of varied H_2_O_2_ concentrations. The bar graphs are presented as the mean ± SD of three independent experiments. ^∗^*p* < 0.05, ^∗∗^*p* < 0.01, ^#^*p* < 0.01 vs. control + CM.

Figure 3 illustrates the findings of the cell migration assay conducted under varying conditions of H_2_O_2_ concentrations and the existence or lack of the hBMSC CM. The *Y*-axis (cell index) represents the extent of cell migration, where higher values indicate greater migration. Several notable observations can be gleaned from the graph. H_2_O_2_ 30% 250 ng/mL + hBMSC CM, depicted in blue, exhibits the most pronounced cell migration throughout the 80-h observation period. A rapid surge in cell migration was observed starting around the 10-h mark, followed by a sustained increase. The green curve represents cells treated with a 30% H_2_O_2_ concentration at 500 ng/mL without hBMSC CM. This treatment also induced a notable cell migration trend, but not as pronounced as the 250 ng/mL with hBMSC CM treatment.

The purple curve represents cell migration under a 30% control with CM (CNT 30% + hBMSC CM). Interestingly, while it started lower than some other conditions, it outpaced several other treatments around the 60-h mark.

H_2_O_2_ 10% conditions curves associated with 10% H_2_O_2_ concentrations (both with and without hBMSC CM) generally display moderate migration rates, with the 500 ng/mL + hBMSC CM curve leading the group.

In differential effects of hBMSC CM, the presence of CM appears to enhance cell migration, especially in the H_2_O_2_ 30% 250 ng/mL and CNT 30% conditions. This suggests a synergistic effect when H_2_O_2_ and hBMSC CM are combined.

Cell migration curves associated with 10% H_2_O_2_ concentrations, both with and without hBMSC CM, generally exhibited moderate migration rates, with the 500 ng/mL + hBMSC CM curve demonstrating the highest migration among these 10% H_2_O_2_ conditions. The presence of hBMSC CM appears to enhance cell migration, particularly in the H_2_O_2_ 30% 250 ng/mL and CNT 30% conditions. This observation suggests a synergistic effect when H_2_O_2_ and hBMSC CM are combined.

### 3.4. Differential Expression of TGF-*β*1, FOS, ATF3, RAF-1, and SMAD3 Genes in HUVECs in Response to Various H_2_O_2_ Concentration Treatment ± hBMSC CM

**Figure 4:**
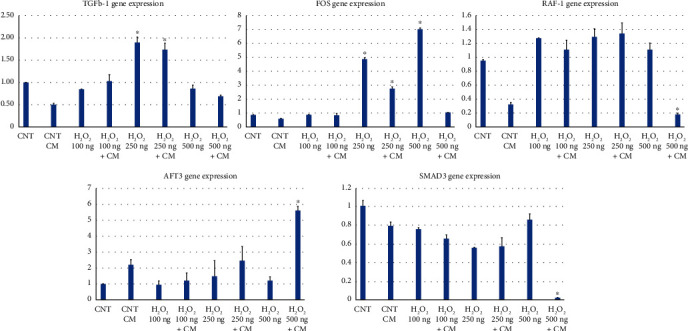
Differential expression of TGF-*β*1, FOS, ATF3, RAF-1, and SMAD3 genes in HUVECs in response to H_2_O_2_ treatment. The bar charts above demonstrate the gene expression levels of TGF-*β*1, FOS, ATF3, RAF-1, and SMAD3 in HUVECs. Each panel represents the relative expression levels of the genes when cells are subjected to various concentrations of H_2_O_2_ treatment with or without hBMSC CM. The control (CNT) serves as a baseline for comparison. The bar graphs are presented as the mean ± SD of three independent experiments. ^∗^*p* < 0.05, ^∗∗^*p* < 0.01 vs. control + CM.

Figure 4 presents bar graphs depicting the relative expression levels of each gene under various conditions. The *Y*-axis represents the fold change in gene expression. There is a notable upregulation of TGF-*β*1 expression under H_2_O_2_ 250 ng + hBMSC CM, indicating that this condition significantly stimulates TGF-*β*1 expression. FOS gene expression is most pronounced under the H_2_O_2_ 500 ng + hBMSC CM. The expression levels of RAF-1 appear relatively consistent across all conditions, with minor variations. The presence of hBMSC CM was able to suppress the RAF-1 expression compared to the H_2_O_2_ alone. There is a sharp spike in ATF3 expression under the H_2_O_2_ 250 ng + hBMSC CM. The expression of SMAD3 is notably upregulated under H_2_O_2_ 500 ng + hBMSC CM.

## 4. Discussion

Inflammation serves as a primary trigger and driving force behind cardiovascular diseases, including endothelial dysfunction and atherosclerosis [2, 3]. Fortunately, MSCs are immunomodulators with therapeutic properties that enable the reduction of chronic inflammation [13–16]. The composition of hBMSC CM is complex and contains a wide array of bioactive factors, including but not limited to growth factors, cytokines, chemokines, and extracellular vesicles. These components can exert profound paracrine effects on target cells, influencing processes such as angiogenesis, apoptosis, proliferation, and migration. For instance, studies have identified specific components within hBMSC CM, such as vascular endothelial growth factor (VEGF), TGF-*β*, and insulin-like growth factor (IGF), that play crucial roles in mediating these protective and restorative effects on endothelial cells [17–19].

In the current work, we used various HUVEC treatment groups in the presence of H_2_O_2_ to depict inflammation. Throughout our experiment, we found (Figure 1) that the proliferation of HUVECs was altered by the presence of H_2_O_2_. Interestingly, this proliferation increase was further enhanced by the combination of various hBMSC CM, which not only demonstrated the ability to protect HUVECs but also exhibited significant effects in promoting HUVEC proliferation even in the presence of H_2_O_2_. Notably, hBMSC CM was able to partially counteract the effects of inflammation. This change is occurring gradually, as evidenced by its onset on Day 2, suggesting that proliferation is slow but effective (Figure 2).

The therapeutic properties of MSCs, particularly their immunomodulatory effects, have been studied extensively for their potential to mitigate chronic inflammation and promote vascular repair [13, 20–22]. Our findings support this potential, indicating that MSCs, through their CM, can enhance endothelial cell survival and proliferation under oxidative stress conditions, which are simulated in this study by H_2_O_2_ treatment.

The expression profiles of TGF-*β*1, FOS, ATF3, RAF-1, and SMAD3 genes in endothelial cells respond dynamically to the inflammatory stimulus of H_2_O_2_, which is modulated by the presence of hBMSC CM. This suggests a complex regulatory network at play, where MSCs might confer protection against endothelial dysfunction through modulation of these critical signaling molecules. Such interactions are pivotal in the endothelium's response to atherogenic stimuli and are increasingly becoming the focus of therapeutic interventions [20, 23].

Previous literature has delineated the roles of the studied genes in vascular biology. For example, TGF-*β*1 is a pleiotropic cytokine involved in endothelial cell function, inflammation, and atherosclerosis [24–26]. Its modulation by MSCs could have implications for plaque stability and the progression of atherosclerosis, where it has been shown to have both atheroprotective and atherogenic effects [26–28]. Likewise, the FOS family of transcription factors, through their role in inflammation, could provide insight into the pathways through which MSCs exert their protective effects in inflammatory vascular diseases [29–32].

Future studies could explore the therapeutic applications of MSCs in clinical settings, considering the insights gained from our gene expression analysis. For example, clinical trials that focus on the use of MSCs for conditions characterized by oxidative stress and inflammation could further validate the potential indicated by our in vitro observations [20, 23, 33]. Moreover, additional bibliographic references can be included that focus on recent advancements in the understanding of MSC-mediated vascular repair mechanisms, the interplay between oxidative stress and endothelial dysfunction, and the translational research bridging these findings with clinical applications [24, 34].

Consistent with our findings, it has been previously established that proliferation occurs as a major physiological homeostatic reaction following inflammation, as proliferation ideally increases the amount of cells required for the restoration of normal physiological conditions [35, 36]. MSCs have been shown in previous studies to diminish inflammation and promote healing in cardiovascular diseases. They exert their therapeutic effects through a variety of mechanisms, including immunomodulation, anti-inflammation, and angiogenesis [6, 33].

Cell migration is a critical process involved in tissue formation, homeostasis, immune response, and wound healing [20]. In the current study, we found that exposure to various concentrations of H_2_O_2_ (mimic inflammation) decreased the HUVEC migration. The presence of hBMSC CM was able to possibly reverse these effects. The H_2_O_2_ 30% 250 ng/mL + hBMSC CM condition induced the most pronounced cell migration, highlighting its potential to stimulate cellular motility. Future research should delve into how hBMSC CM positively impacts the process of cellular migration. Indeed, cell migration is significantly influenced by alterations in cellular metabolism. Metabolic changes can activate cellular signaling pathways, triggering a cascade of events that promote cell movement [20].

MSCs have the potential to migrate to the site of proliferation and restore physiological conditions by reactivating immune responses [23]. A study utilizing a fetal sheep model demonstrated that human MSCs possess the ability to reprogram and differentiate into tissue-specific cells in noninjury settings [34]. However, there remains a paucity of evidence elucidating the mechanisms by which MSCs reprogram the genetic landscape to alter cellular fate [23]. In our study, we analyzed the genetic expression of five inflammation-related genes: TGF-*β*1, FOS, RAF-1, ATF3, and SMAD3. We observed alterations in gene expression upon exposure to varying concentrations of H_2_O_2_, both with and without hBMSC CM. The most notable gene expression change under the H_2_O_2_ 500 ng + hBMSC CM condition was the upregulation of TGF-*β*1.

TGF-*β*1 is a cytokine with a complex and multifaceted role in inflammation. It can act as both a proinflammatory and anti-inflammatory mediator. TGF-*β*1 is recognized as the master regulator of numerous biological processes due to its involvement in cell proliferation, migration, apoptosis, differentiation, autophagy, and, ultimately, immune responses [24]. TGF-*β*1 can promote inflammation by inducing inflammatory mediators, enhancing immune cell migration and activation, and promoting proinflammatory T helper cells. It also suppresses inflammation by inducing apoptosis of activated immune cells, promoting regulatory T cell differentiation, inhibiting inflammatory mediators, and promoting tissue repair [25]. The graph (Figure 4) suggests that TGF*β*-1 expression is induced by H_2_O_2_, with higher levels at increased concentrations. However, in the presence of hBMSC CM, the expression seems to be modulated, suggesting a potential protective effect of hBMSC CM against oxidative stress-induced expression of TGF*β*-1. This modulation emphasizes the dual role of TGF-*β*1 in endothelial dysfunction, atherosclerosis, and plaque stability [27]. Also, TGF-*β*2 was found to be the most prevalent form in human atherosclerotic plaques. This isoform is thought to promote plaque stability by reducing inflammation and matrix breakdown [28].

The FOS family of transcription factors plays a crucial role in regulating inflammation by activating genes that promote inflammation. FOS is rapidly induced in response to inflammatory stimuli and binds to the AP-1 transcription factor, leading to the expression of proinflammatory cytokines, adhesion molecules, and iNOS [29]. FOS proteins have been associated with the progression of inflammatory diseases like rheumatoid arthritis, inflammatory bowel disease, endothelial dysfunction, and atherosclerosis [30, 32]. In the present study, exposure of HUVECs to H_2_O_2_ led to a significant increase in FOS expression, which was reversed when cells were cultivated in the presence of hBMSC CM. This suggests a potential positive role for hBMSC CM in HUVEC survival and stress response regulation.

RAF-1 is a central component of the MAPK/ERK signaling pathway. By activating MAPK/ERK signaling, interacting with inflammatory proteins, and modulating cell migration, RAF-1 contributes to the inflammatory response, cell division, differentiation, and survival [37]. RAF-1 expression was significantly suppressed when HUVECs were cultivated with H_2_O_2_ 500 ng + hBMSC CM. The varying levels of RAF-1 expression exhibited by HUVECs across H_2_O_2_ concentrations could reflect a complex regulatory mechanism in which hBMSC CM may help stabilize the cell's response to oxidative stress, potentially beneficial for maintaining endothelial function during endothelial dysfunction and atherosclerotic stress.

ATF3, an immunomodulator transcription factor within the ATF/CREB family, plays a dual role in inflammation, acting as both a proinflammatory and anti-inflammatory mediator. ATF3 can suppress inflammation by inducing apoptosis of activated immune cells, promoting regulatory T cell differentiation, inhibiting inflammatory mediator production, and facilitating damaged tissue repair [38]. The ATF3 role in endothelial dysfunction and atherosclerosis is controversial [39]. In the present study, the substantial upregulation of ATF3 expression by HUVECs at the high H_2_O_2_ concentration with hBMSC CM suggests a robust stress response, potentially alleviated by hBMSC CM. This could indicate an enhanced protective stress response that might be beneficial for cells under oxidative stress conditions relevant to endothelial dysfunction and atherosclerosis. These findings corroborate previous research demonstrating a protective role for ATF3 in regulating macrophage lipid metabolism, further establishing ATF3 as a critical nexus for integrating lipid metabolic and inflammatory signaling pathways in these cells, thereby potentially mitigating atherosclerosis development [40, 41].

SMAD3 gene is involved in the TGF-*β* signaling pathway and plays a role in the regulation of cellular proliferation and apoptosis [42]. The decrease in expression of SMAD3 with higher H_2_O_2_ concentrations could suggest a toxic effect that overwhelms the cell's protective responses, or it could be indicative of a negative feedback mechanism. The presence of hBMSC CM might be providing a counterbalancing effect, perhaps indicating a role in protecting endothelial cells from the adverse effects of oxidative stress.

## 5. Conclusion

Our study harnesses the immunomodulatory prowess of MSCs, which have shown promise in decreasing chronic inflammation associated with atherosclerosis. CM not only preserves but enhances the proliferation of endothelial cells even under oxidative stress, as marked by H_2_O_2_ exposure. Moreover, our study delves into the molecular realm, highlighting significant changes in gene expression levels pertinent to inflammation and cellular repair mechanisms. Notably, the upregulation of TGF-*β*1 in the presence of CM signifies its crucial role as a regulatory element in immune responses and tissue homeostasis. Furthermore, the study demonstrates that MSC-CM promotes cellular migration, a crucial step in tissue regeneration, potentially through modulating cellular signaling and metabolism. These insights build upon existing literature, reinforcing the therapeutic potential of MSC-CM in combating neuroinflammation, and further broadening its application scope.

Our research substantiates the therapeutic value of hMSC-CM, setting a precedent for its application in mitigating oxidative stress–related endothelial dysfunction. It lays a foundation for future in vivo studies, which could translate these promising in vitro results into novel clinical interventions for cardiovascular disease treatment. To support these data, it would be appropriate to validate the results by analyzing HUVEC viability, proliferation, and migration with other methods.

## Figures and Tables

**Table 1:** Different HUVEC study groups.

**Groups**	**Investigated groups**
1	HUVECs cultured alone (control group; CNT)
2	HUVECs cultured with H_2_O_2_ 100 ng/mL
3	HUVECs cultured with H_2_O_2_ 250 ng/mL
4	HUVECs cultured with H_2_O_2_ 500 ng/mL
5	HUVECs cultured with hBMSC CM (CNT + CM)
6	HUVECs cultured with H_2_O_2_ 100 ng/mL + hBMSC 30% CM
7	HUVECs cultured with H_2_O_2_ 250 ng/mL + hBMSC 30% CM
8	HUVECs cultured with H_2_O_2_ 500 ng/mL + hBMSC 30% CM

Abbreviations: CM: conditioned media, hBMSCs: human bone marrow–derived mesenchymal stem cells, HUVECs: human umbilical vein endothelial cells.

**Table 2:** Primers used in the RT–PCR.

**Gene**	**Primer**	**Primer sequence**
TGF-*β*1	Forward	5′-GGACCTCGCAACAACGTCGCTG-3′
	Reverse	5′-TGGTAGACGATCAGACGTCTC-3′

FOS	Forward	5′-GCCTCTCTTACTACCACTCACC-3′
	Reverse	5′-AGATGGCAGTGACCGTGGGAAT-3′

ATF3	Forward	5′-AACCTCATGGGTTCTCCAGCGA-3′
	Reverse	5′-CTCCAACATCCAATCTGTCCCG-3′

RAF-1	Forward	5′-TGTCAGCGTCATGGCTCCAG-3′
	Reverse	5′-CAGCCTCCTTCCATGTCTCC-3′

SMAD3	Forward	5′-TGAGGCTGTCTACCAGTTGACC-3′
	Reverse	5′-GTGAGGACCTTGTCAAGCCACT-3′

GAPDH	Forward	5′-GAAGGTGAAGGTCGGAGTC-3′
	Reverse	5′-GAAGATGGTGATGGGATTTC-3′

## Data Availability

All data are included in the manuscript.

## Nomenclature

*ATF3*: activating transcription factor 3, proto-oncogene
cDNA: complementary deoxyribonucleic acid
CM: conditioned media
CNT: control group
DMEM: Dulbecco Modified Eagle Medium
DNA: deoxyribonucleic acid
ERK: extracellular regulated kinase
*FOS*: proto-oncogene, AP-1 transcription factor subunit
H_2_O_2_: hydrogen peroxide
hBMSCs: human bone marrow–derived mesenchymal stem cells
HUVECs: human umbilical vein endothelial cells
iNOS: inducible nitric oxide synthase
MAPK: mitogen-activated protein kinase
MSCs: mesenchymal stem cells
PBS: phosphate-buffered solution
*RAF-1*: accelerated fibrosarcoma-1
RT–PCR: real-time polymerase chain reaction
*SMAD3*: suppressor of mothers against decapentaplegic 3
*TGF-β1*: transforming growth factor-beta 1
